# Clinical and biological effects of high-dose sodium selenite, continuously administered in septic shock

**DOI:** 10.1186/cc10385

**Published:** 2011-10-27

**Authors:** X Forceville, D Vitoux, W Wasowicz, M Dehoux, P Van Antwerpeen, D Annane, E Plouvier, A Boutten, J Gromadzinska, B Laviolle, A Combes, E Bellissant

**Affiliations:** 1 Réanimation Polyvalente, CH de Meaux, France; 2 Biochimie A, CHU St Louis, Paris, France; 3 Toxicology and Carcinogenesis, Nofer Institute, Lodz, Poland; 4 Biochimie métabolique et cellulaire, CHU Bichat, Paris, France; 5 Chimie pharmaceutique organique, Université Libre de Bruxelles, Belgium; 6 Réanimation, CHU Poincaré, Garches, France; 7 Biochimie CH de Meaux, France; 8 Recherche - Santé Publique, CIC Inserm 0203, CHU Pontchaillou, Rennes, France

## Introduction

Sodium selenite (Na_2_SeO_3_) has been proposed as an early treatment of septic shock with discrepant results [1-3]. Beneficial action is mainly believed through improvement of major antioxidant selenoenzymes, but could on the contrary be related to a therapeutic oxidant action reducing activity of hyperactivated circulating phagocytic cells [4]. It has been suggested that the absence of beneficial effect of high-dose Na_2_SeO_3 _continuously administered [2] might be related to toxicity, especially on the lung, of too much selenium (Se) as mentioned in recent parenteral nutrition guidelines in intensive care [5]. On additional clinical and biological data, our purpose was to assess if there was argument for Na_2_SeO_3 _toxicity, especially on the lung, under continuous administration of high-dose Na_2_SeO_3 _in the SERENITE study.

## Methods

In a randomized, double-blind multicenter study performed in 60 septic shock patients [2], the efficacy and tolerance of Na_2_SeO_3 _(4 mg Se on day 1 (D1), then 1 mg/day during 9 days or placebo) were evaluated on all components of the SOFA score measured daily, infection rate, and plasma Se, selenoprotein-P (Sel-P), glutathione peroxidase (GPx), lipid peroxidation, cytokines, and procalcitonin measured at D0, D4, D7, D10 and D14.

## Results

**Figure 1:**
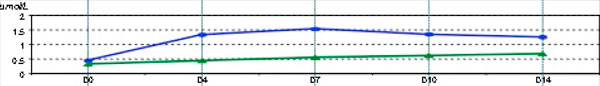
**Plasma Se concentration according to groups and time**. Data presented as mean ± SD (μmol/l). Reference value for plasma Se concentration: 1 ± 0.15 μmol/l. Treated group indicated in blue and nontreated in green.

No deleterious effect of Na_2_SeO_3 _especially on the lung was observed for any clinical or biological variables. PaO_2_/FiO_2 _was strictly identical between groups (Table 1). As compared with placebo, mean time occurrences of infections were delayed in the treated group (18 ± 24 days vs. 34 ± 28 days, respectively; *P *< 0.0001). Plasma Se, Sel-P and GPx concentrations were increased at D4 in the treated group, achieving the high reference value for the plasma Se concentration (Figure 1).

**Table 1:** PaO_2_/FiO_2 _according to group and time

	Baseline (D0)	D2	D3	D4	D7	D10	D14
Na_2_SeO_3_	20 ± 15	23 ± 12	24 ± 10	25 ± 14	26 ± 11	30 ± 13	28 ± 15
Placebo	22 ± 13	21 ± 11	25 ± 13	26 ± 11	28 ± 14	33 ± 17	36 ± 17
*P *value	0.60	0.37	0.92	0.81	0.65	0.52	0.13

Data presented as mean ± SD (KPa).

## Conclusion

Continuous administration of high doses of Na_2_SeO_3 _(4 mg Se D1) did not induce any deleterious effect in septic shock patients. We did not observe a beneficial effect, contrasting with a comparable study administering Na_2_SeO_3 _in bolus, potentially more toxic [1]. In agreement with results obtained on a peritonitis sheep model [6], our data support a therapeutic oxidant action of Na_2_SeO_3_, opening a new field in septic shock treatment based on oxidant selenocompounds.
